# Severe irritant contact dermatitis induced by povidone iodine solution

**DOI:** 10.4103/0253-7613.56069

**Published:** 2009-08

**Authors:** Mangala Bhaskar Murthy, Bhaskar Krishnamurthy

**Affiliations:** Departments of Pharmacology, Obstetrics and Gynecology, GMC, Miraj, India

**Keywords:** Chemical burns, irritant contact dermatitis, povidone iodine

## Abstract

Albeit uncommon occurrence, irritant contact dermatitis induced by povidone iodine can be an unfortunate adverse reaction complicating its use as an antiseptic. We hereby present the case report of a patient who suffered such a reaction as a result of exposure to povidone iodine, employed as an antiseptic during spinal anesthesia. On conservative management with soframycin ointment, the lesions resembling chemical burns healed in a month without extensive scarring or other complications.

## Introduction

Use of iodine as an antiseptic has gone into disrepute as a result of its short lasting action and irritant properties. Strong solution of iodine is corrosive and can cause blistering and necrosis of skin, commonly referred to as chemical burns or irritant contact dermatitis. Iodine has thus been replaced by substances known as iodophores that contain an iodine molecule linked to a large molecular-weight organic compound. A slow release of iodine from the polymer contributes to a long lasting action and a low irritant profile of such compounds. Povidone iodine is one such iodophore that has poor irritant property despite preserved antiseptic efficacy, and hence is advocated as a non-irritant, non-toxic compound for surgical scrubbing and disinfection of endoscopes.

Here, we report an unfortunate case of severe irritant contact dermatitis caused by the so-called, non-irritant compound.

## Case Report

**Figure 1:**
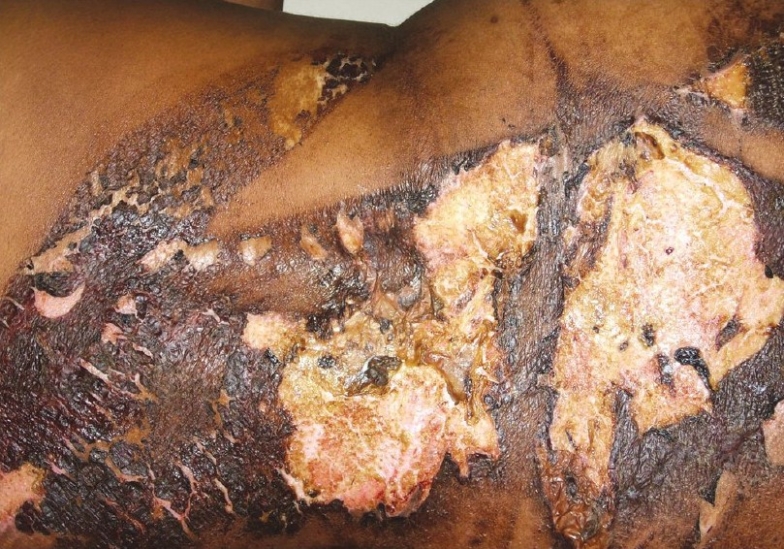
Macerated skin on the back as a result of exposure to povidone iodine

A 28-year-old full-term pregnant woman was posted for emergency caesarean section under spinal anesthesia after eliciting a short history of drug allergy that was found to be negative. The surgery was uneventful. On the first post-operative day, the patient complained of burning sensation on the back. The examination of the back revealed complete erythema of the back extending from the subscapular region to the buttocks corresponding to the area painted by 10% povidone iodine solution as an antiseptic for administration of spinal anesthesia. By the second post-operative day, the whole region turned brownish black in colour and progressed to blistering and patchy skin necrosis, and exfoliation by the fifth post-operative day [Figure 1].

The general examination of the patient revealed no abnormality. Body temperature was normal, and unexposed skin and the mucous membrane were completely spared. Characteristic features of the lesion like the localization to the area of exposure to povidone iodine, extension of lesions to the region of skin folds along the flank due to gravitation of solution during the process of painting served as strong factors in supporting the diagnosis of povidone iodine-induced irritant contact dermatitis. Thereafter, the patient was managed by daily dressing with soframycin skin ointment, and analgesics as and when required. Steroids were deliberately avoided considering their tendency to promote thinning of skin and necrosis in addition to increasing the risk of infection, both of which could worsen the patient's condition. Despite lesions corresponding to 9% second and third degree chemical burns, the patient did not require any intensive treatment or surgical intervention during the follow-up, and lesions healed without extensive scarring or other complications.

## Discussion

Even though povidone iodine is classified as a non-irritant antiseptic, it is not completely devoid of corrosive action. Although the present case is one of the severest of its kind to be reported, some journals have reported similar cases of chemical burns induced by povidone iodine in patients undergoing surgeries and arteriographies.[1–3] A similar case report of povidone iodine-induced chemical burns states that the danger of such burns may be exaggerated with the use of an outdated povidone iodine solution,[4] although it was not so in our case. Burns of this kind, however, occur mostly in patients undergoing uro-gynecological surgeries, in which lithotomy position favors irritation, maceration, and necrosis of pressure points on the skin, like the back in this case.[5] Hence, irritant contact dermatitis should always be considered as a differential diagnosis in patients exposed to povidone iodine, developing lesions resembling burns, and in spite of an uncommon occurrence, stress has to be laid on the fact that this condition can be effortlessly prevented by just allowing the solution to dry before the patient is draped to avoid long contact period and by checking for the expiry date on the label of the povidone iodine solution.

## References

[1] Nahlieli O, Baruchin AM, Levi D, Shapira Y, Yoffe B (2001). Povidone-iodine related burns. Burns.

[2] Demir E, O'Dey DM, Pallua N (2006). Accidental burns during surgery. J Burn Care Res.

[3] Liu FC, Liou JT, Hui YL, Hsu JC, Yang CY, Yu HP (2003). Chemical burn caused by povidone iodine alcohol solution: A case report. Acta Anaesthesiol Sin.

[4] Kara A, Tezer H, Devrim I, Cengiz AB, Secmeer G (2007). Chemical burn: A risk with outdated povidone iodine. Pediatr Dermatol.

[5] Hodgkinson DJ, Irons GB, Williams TJ (1978). Chemical burns and skin preparation solutions. Surg Gynecol Obstet.

